# Laparoscopic vs. Robotic transabdominal adrenalectomy- propensity matched analysis and learning curve

**DOI:** 10.1007/s00423-025-03688-7

**Published:** 2025-03-27

**Authors:** M. Meir, A. Wiegering, F. Sperschneider, A. Hendricks, J. F. Lock, S. Flemming, M. Kelm, S. Hahner, N. Schlegel, C. T. Germer, J. Reibetanz

**Affiliations:** 1 Department of General-, Visceral-, Transplant-, Vascular- and Pediatric Surgery, University Hospital of Wuerzburg, Wuerzburg, Germany; 2 Department of Medicine, Division of Endocrinology and Diabetes, University Hospital Wuerzburg, Wuerzburg, Germany

**Keywords:** Laparoscopic, Robotic, Adrenalectomy, Transabdominal, Learning curve

## Abstract

**Background:**

While laparoscopic transabdominal or retroperitoneal adrenalectomy is standard care for adrenal tumors, benefits of robotic adrenalectomy (RA) are yet unclear. We evaluated the costs (including disposables), outcomes as well as the learning curve of robotic and laparoscopic adrenalectomy (LA) in a specialized center.

**Methods:**

In retrospective analysis of our prospective database (ethical approval number 88/11) 263 LA were compared to 27 RA in the study period between 2018 and 2023. A propensity score match analysis was used to exclude possible confounders. Furthermore, the learning curve of RA was investigated.

**Results:**

Intraoperative Riva Rocci (RR) fluctuations (> 160mmHg, < 90 mmHg), early complications (within 30 days) and intraoperative blood loss were comparable in both groups. However, length of stay was decreased following robotic adrenalectomy (3.50d ± 1.81d compared to 4.61d ± 2.75d; *p* = 0.04). Due to this, overall costs of RA were lower compared to LA even if cost for disposables were slightly higher in the robotic group. An analysis of the learning curve of robotic adrenalectomy revealed that learning curve is completed after 5- 6th procedure.

**Conclusions:**

Taken together our study supports the fact that RA is as secure and feasible as LA. Furthermore, it might provide advantages due to shorter length of stay, a short learning curve and similar costs compared to LA.

## Introduction

While LA is the standard of care for small adrenal tumors [1–4], more and more specialized centers have switched to RA in recent years [5]. The widespread availability of robotic devices has encouraged centers to take advantage of the benefits of robotic surgery, such as the ability to perform precise movements in a limited working space, the reduction of tremor, orientation errors due to camera fixation, three-dimensional optics with a higher resolution and stereopsis, and of course the ergonomic benefits for the surgeon [6, 7]. However, RA has yet failed to show clear improvements with regard to estimated blood loss, conversion rate or complication rates compared to LA, while costs suggested to be higher than laparoscopic surgery [8]. To date, there are only a few studies with small patient cohorts, thus, overall evaluation of possible advantages of RA remain unclear. A recent meta-analysis [5] compared 747 RA to 415 LA and did not report a significant difference in conversion rate, estimated blood loss, morbidity and mortality with a slight shorter hospital stay of 0.5 days (2.88 d after LA comparted to 2.38 days after RA) and a longer operation time. The authors concluded that RA is a safe and effective procedure, but more studies are needed to discriminate the differences.

We changed our own standard to RA in May 2023 and wanted to analyze costs (including disposables), outcomes as well as learning curves of RA compared to LA after one year.

## Materials and methods

### Study design

The local ethics committee in Wuerzburg approved the study (No 88/11). Patients undergoing minimal invasive LA or RA (daVinci XI system) at the Department of Surgery, University Hospital Wuerzburg, between January 2018 and December 2023 were recruited from our prospective database (ENSAT register). Bilateral LA or RA were excluded. With 60 minimal invasive adrenalectomies performed per year the university hospital is regarded as specialized, high volume center [9]. All patients were divided into two groups according to the type of surgery (LA versus RA). Baseline patient’s characteristics included age, body mass index, (BMI), gender, the Charlson comorbidity Index, tumor side localization, hormonal status, tumor size and prior abdominal surgery. The operations were performed by two robotic-experienced surgeons (> 50 robotic procedures) and experienced surgeons in LA (> 100 procedures).

In addition, we analyzed the rate of conversion, operating time as well as complications according to the Clavien-Dindo classification.

Perioperative parameters included, intraoperative RR fluctuations (> 160mmHg, < 90 mmHg), length of stay, early complications (within 30 days), postoperative C- reactive protein levels on the first and third and postoperative day. Furthermore, the difference in hemoglobin levels on the preoperative day and on the first day after the operation to validate intraoperative blood loss and mortality (within 90 days) were recorded.

### Cost parameters

In order to analyze the expenditure for each procedure, cost parameters were collected by the controlling department. These included the cost of consumables (surgical equipment such as robotic instruments, amortization of robotic and laparoscopic equipment, ultrasound dissectors, drapes, sutures and sterile supplies) and hospital-related costs (e.g. the charge per minute of surgery, the cost of a day in intensive care or a day on the regular ward).

### Propensity matching

To exclude possible confounders of the results, we used a propensity score matching analysis for lesion size and tumor localization, hormonal secretion status, BMI, and previous abdominal surgery.

### Statistical analysis

We used IBM SPSS 28.0 (IBM SPSS, USA) for statistical analysis. Chi²-test (indicated by * in the tables) as well as single factor variance were used to detect differences between LA and RA. Significance was assumed for *p* < 0.05. Descriptive analyses included mean (MV), minimum (min), and maximum (max) values given as range, percentage, and standard deviation.

## Results

### Baseline characteristics

During the study period (2018 and 2023), 313 patients underwent minimal invasive transabdominal adrenalectomy. 23 bilateral adrenalectomies were excluded. Of the remaining 290 patients, 263 underwent LA and 27 RA. In the first years, all patients underwent LA, while RA was introduced in May 2023, with most of the patients receiving robotic-assisted surgery for the rest of the observational period. Baseline characteristics of laparoscopic operated patients (*n* = 263) and robotic patients (*n* = 27) are comparable and shown in Table 1. Although there were significantly more women (23/27 (85.2%) in RA compared to 156/263 (59.3%) in LA) and tumor size was larger in the laparoscopic group (3.5 cm compared to 2.5 cm), other relevant factors such as age, BMI, Charlson comorbidity index, hormone secretion and previous abdominal surgery did not differ.

**Table 1:** Baseline characteristics

Characteristics	Laparoscopic *n*= 263	Robotic *n*= 27	*p*-value
Sex			0.04*
Male n (%)	113 (42.0%)	4 (14.8%)	
Female n (%)	156 (58.0%)	23 (85.2%)	
Age at operation [years] mean (range)	51.0 (4- 80)	55.3 (27 -74)	0.12
BMI [kg/m^2^] mean (range)	29.8 (14.9- 47.1)	29.4 (17.6-46.1)	0.83
ASA classification mean (range)	2.26 (1-4)	2.2 (1-3)	0.48
Charlson Comorbidity Index mean (range)	2.27 (1.9 - 2.6)	2.73 (1.63 – 3.83)	0.36
Tumor side localization			0.87*
Right	142	11	
Left	121	16	
Hormone secretion			0.175*
None	61	8	
Pheochromocytoma	51	1	
Conn’s	88	8	
Cushing’s	65	10	
DHEA	4	0	
Tumor size [cm] (± SD)	3.50 (± 2.13)	2.50 (± 1.81)	**0.03**
Prior abdominal Operation			0.157*
None	167	16	
Laparoscopic	41	4	
Laparotomy	61	7	

### Perioperative results of RA and LA

**Fig. 1:**
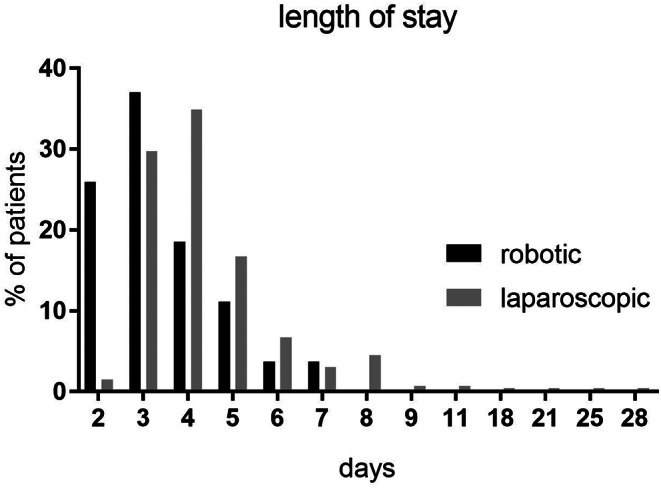
Comparison of length of stay between RA and LA is shown. On average, patients stayed 4.61d ± 2.75d after LA compared to 3.50d ± 1.81d after RA

The perioperative outcomes of patients who underwent LA or RA were analyzed and are shown in Table 2. Perioperative morbidity and operation time did not differ in RA, though operation time was slightly longer in LA (72.39 ± 25.65 min in the laparoscopic vs. 81.50 ± 52.60 min in the robotic group; *p* = 0.12). In addition, perioperative markers such as C- reactive protein or hemoglobin levels (as a marker of perioperative blood loss) did not show significant different gradients, nor relevant fluctuations in RR were observed. However, length of stay (3.50d ± 1.81d) and stay on intensive care (0.41d ± 0.58d) was significantly shorter in RA compared to LA (4.61d ± 2.75d length of stay and 1.0d ± 1.79d stay on intensive care; *p* = 0.04) (Fig. 1).

**Table 2:** Perioperative outcomes

Perioperative outcomes	Laparoscopic *n*= 263	Robotic *n*= 27	*p*-value
Length of stay [days]	4.61 d (± 2.75)	3.50 d (± 1.81)	**0.04**
Days on ICU [days]	1.0 (± 1.79)	0.41 (± 0.58)	**0.01**
Operation time [minutes] (± SD)	72.39 (± 25.65)	81.50 (± 52.60)	0.12
Complications according to Clavien Dindo			0.59*
I	11	0	
II	4	0	
IIIa	1	0	
IIIb	1	1	
IV	0	0	
Comprehensive Complication Index (± SD)	3.34 (± 9.40)	4.06 (± 9.50)	0.14
Re – Operation (within 30d)	3	1	0.22
Mortality (within 90d)	0	0	
Wound infection (within 30d)	1	0	0.68
Conversion to laparotomy	2	0	0.54
Malignant tumor	25	4	0.25
PreOP hemoglobin level [g/dl] (± SD)	13.8 (±1.5)	13.7 (±1.6)	0.94
PostOP hemoglobin level [g/dl] (± SD)	12.6 (±1.7)	12.2 (±1.5)	0.29
Hemoglobin Difference [g/dl] (± SD)	1.30 (± 1.18)	1.52 (± 1.54)	0.52
Intraoperative blood transfusion	0	0	0.11
CRP level 1^st^ postOP day [mg/dl] ± SD)	1.84 (± 1.50)	1.99 (± 1.40)	0.15
CRP Level 2^nd^ postOP day [mg/dl] (± SD)	3.4 (± 3.92)	5.62 (± 7.62)	0.62
RR <90mmHg / >160mmHg intraOP	24	4	0.14
Cost of intraoperative medical disposables (including amortization of robotic and laparoscopic equipment)	608.86 €	713.76 €	
Total Costs (including length of stay)	4,536.35 €	3,466.36 €	

An overall low comprehensive complication index according to the Clavien-Dindo classification with 4.65 ± 13.0 points in the laparoscopic group compared 5.84 ± 12.9 points in the robotic group revealed that minimal invasive adrenalectomy is a surgical safe procedure with an overall low morbidity.

None of the patients died within 90 days after surgery. Three patients in the laparoscopic (1.9%) and one patient (3.7%) in the robotic group were reoperated due to postoperative hematoma or bleeding. Furthermore, two patients were converted to open surgery in the LA and none in RA.

### Propensity matched analysis of laparoscopic and robotic adrenalectomy

To exclude possible confounders on outcomes, we used a propensity score matching analysis for lesion size and tumor localization, hormonal secretion status, BMI, and prior abdominal surgery (Table 3).

**Table 3:** Propensity match of perioperative outcomes

Perioperative outcomes	Laparoscopic *n*= 27	Robotic *n*= 27	*p*-value
Length of stay [days]	5.35 (± 5.70)	3.50 d (± 1.81)	**0.01**
Days on ICU [days]	1.51(± 1.92)	0.41 (± 0.58)	**0.001**
Operation time [minutes] (± SD)	83,90 (± 33,18)	81.50 (± 52.60)	0.91
Complications according to Clavien Dindo			0.55*
I	1	0	
II	1	0	
IIIa	1	0	
IIIb	1	1	
IV	0	0	
Comprehensive Complication Index time (± SD)	7.88 (± 13.7)	4.06 (± 9.50)	0.34
Re – Operation (30d)	1	1	0.22
Mortality (90d)	0	0	
Wound infection (30d)	1	0	0.68
Conversion to laparotomy	0	0	0.54
Malignant tumor	4	4	0.25
PreOP hemoglobin level [g/dl] (± SD)	13.8 (±1.6)	13.7 (±1.6)	0.94
PostOP hemoglobin level [g/dl] (± SD)	12.2 (± 1.6)	12.2 (±1.5)	0.29
Hemoglobin Difference [g/dl] (± SD)	1.38 (± 1.12)	1.52 (± 1.54)	0.52
Intraoperative blood transfusion	0	0	0.11
CRP level 1^st^ postOP day [mg/dl] (± SD)	1.92 (± 1.36)	1.99 (± 1.40)	0.15
CRP Level 2^nd^ postOP day [mg/dl] (± SD)	3.88 (± 5.77)	5.62 (± 7.62)	0.62
RR <90mmHg / >160mmHg intraOP	4	4	0.15

This propensity-matched analysis revealed that there were no significant differences between LA and RA. In contrast, the trend of a shorter operation time in LA was even less pronounced if prior operations, tumor size, localization and BMI were taken into account with an operation time of 83.90 ± 33.18 min in LA compared to 81.50 ± 52.60 min in RA. Furthermore, the significant shorter length of stay was even more relevant in the propensity matched group, where laparoscopic patients stayed 2 days longer than the robotic patients.

### Learning curve of RA

**Fig. 2:**
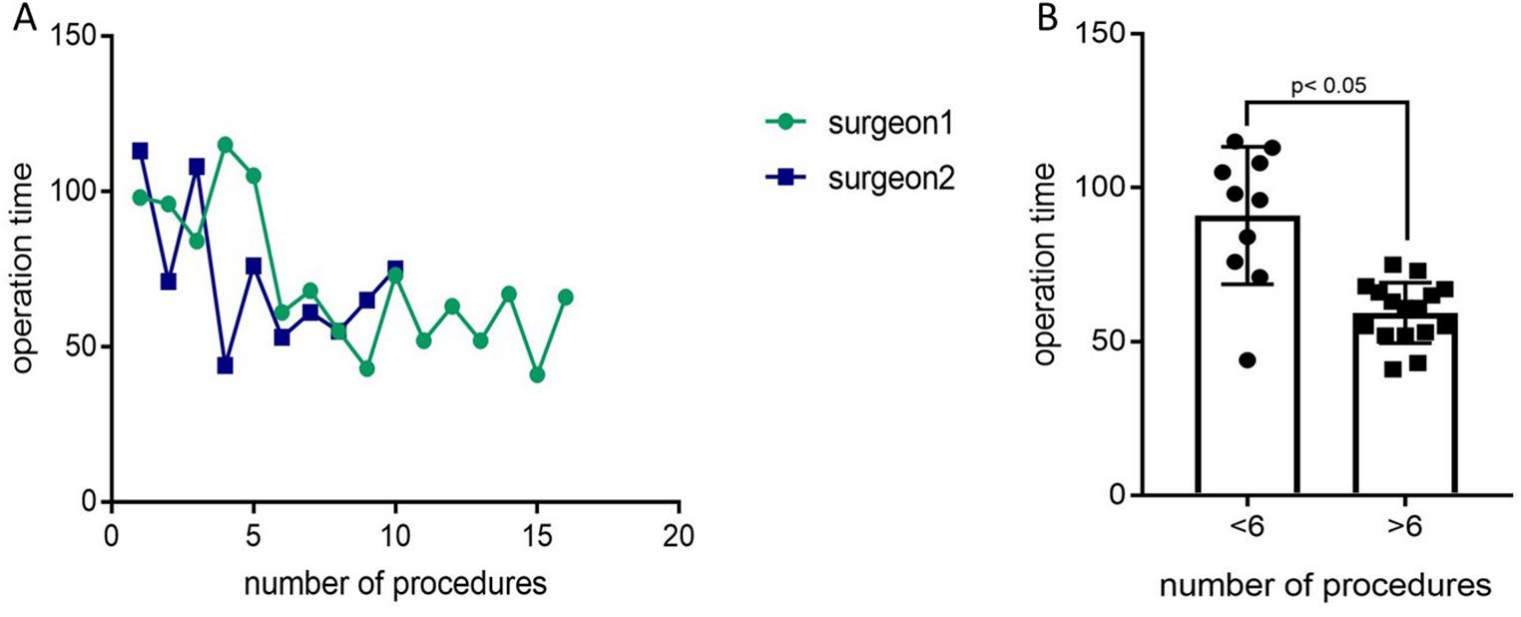
**A**: The learning curve of the two surgeons is shown with the operative time for each procedure. **B**: A comparison of the operative time showed that the operative time decreased from 91 ± 22.3 min to 59.4 ± 9.8 min after the 6th surgery

The learning curve of RA of the two attending, robotic-experienced surgeons is shown in Fig. 2. The average operation time went down from 91 ± 22.3 min to 59.4 ± 9.8 min after the 6th operation.

### Cost analysis of RA compared to LA

A detailed analysis of the medical disposables used for either LA or RA showed, that disposable materials in RA are slightly more expensive. In LA the overall cost of medical disposables and amortization of instruments is 608.86 € compared to 713.76 € in robotic surgery. But due to the shorter length of stay over all costs are in total in favor of robotic surgery where average costs went down to 3466.36 € compared to 4536.35 € in LA.

## Discussion

Minimal invasive, LA is the surgical standard of care for adrenal tumors. While to date LA is well established to date, evidence on RA remain limited and important questions unanswered. Here we provide a substantial verification on the robotic approach in a large patient cohort of patients including propensity score matching. Based on that, our study demonstrates that both RA and LA are safe and feasible surgical procedures in a referral center but length of stay is shorter in RA.

Although in our cohort RA did not show a distinctive advantage in our cohort, there might be benefits that should be considered. Firstly, in the long term the operation time might be shorter in RA. The duration of a RA after the learning curve was significantly shorter compared to LA. This is in line with other groups, who have also reported a shorter operative time [10]. This fact, in contrast to many other robotic procedures, might be easily explained by the ability to articulate the instruments and the use of a fourth arm for additional counter traction especially on the right side with limited retroperitoneal space.

The relatively short learning curve of RA must also be taken into account. In LA, a significantly shorter operative time has been reported after 30 to 40 procedures, while other papers have shown a similarly short learning curve in RA compared to our study [4–7]. However, our results may be biased because the surgeons were experienced in both LA (more than 100 adrenalectomies per surgeon) and robotic surgery (more than 50 robotic procedures per surgeon). Experienced surgeons may adapt quickly to RA, but inexperienced surgeons may have difficulty overcoming the learning curve.

At last costs must be considered. Robotic systems might have limitation, since acquisition and maintaining costs are high, however, careful evaluation of its financial implications is needed. While other groups postulated that RA has lower complication rates compared to LA [11–13], they concluded that despite the higher costs of RA is cost-effective and economically sustainable in high-volume centers especially when performed for challenging cases [14, 15].

Similarly, our cost analysis showed that robotic surgery is only slightly more expensive than laparoscopic surgery when using an ultrasonic dissection device for LA. Furthermore, when total hospital costs are considered, RA is cost effective due to the shorter length of stay.

In conclusion, our study supports the fact that RA is as safe and feasible as LA. It may also have advantages over laparoscopic adrenalectomy in terms of shorter length of stay, shorter learning curve and similar costs.

## Strengths and limitations

The present study has limitations. First, the study was conducted in a referral centre for adrenal pathologies. Second, the study was retrospective. However, the strengths of the study are the large sample size for a rare disease and the large collection of clinical data.

## Data Availability

No datasets were generated or analysed during the current study.
